# RPS23RG1 inhibits SORT1-mediated lysosomal degradation of MDGA2 to protect against autism

**DOI:** 10.7150/thno.100451

**Published:** 2025-01-01

**Authors:** Yuanhui Huo, Dongdong Zhao, Xiang Zhu, Naizhen Zheng, Dingting Yang, Jian Meng, Yiqing Chen, Yun-wu Zhang

**Affiliations:** 1 Xiamen Key Laboratory of Brain Center, The First Affiliated Hospital of Xiamen University, and Fujian Provincial Key Laboratory of Neurodegenerative Disease and Aging Research, Institute of Neuroscience, School of Medicine, Xiamen University, Xiamen, China.; 2 Institute of Aging, Key Laboratory of Alzheimer's Disease of Zhejiang Province, Wenzhou Medical University, Wenzhou, Zhejiang 325035, China.

**Keywords:** autism spectrum disorder, MDGA2, lysosomal degradation, RPS23RG1, SORT1

## Abstract

**Rationale:** Mutations in the synaptic protein MAM domain containing glycosylphosphatidylinositol anchor 2 (MDGA2) have been associated with autism spectrum disorder (ASD). Therefore, elucidating the regulatory mechanisms of MDGA2 can help develop effective treatments for ASD.

**Methods:** Liquid chromatography-tandem mass spectrometry was carried out to identify proteins interacting with the extracellular domain of RPS23RG1 and with MDGA2, followed by co-immunoprecipitation assays to confirm protein-protein interactions. RPS23RG1 and SORT1 levels were downregulated by siRNAs to study their effects on MDGA2 degradation, with additional applications of immunoblotting and immunostaining assays. Lysosome isolation was performed to determine the lysosomal degradation of MDGA2 further. *Rps23rg1* knockout mice and *Mdga2*^+/-^ mice were subjected to various behavioral tests to study their ASD-like phenotypes. AAVs expressing MDGA2 were delivered in *Rps23rg1* knockout mice, and RPS23RG1-derived peptide was delivered in *Mdga2*^+/-^ mice to study their rescuing effects.

**Results:** We found that both RPS23RG1 and SORT1 interacted with MDGA2. MDGA2 was primarily degraded through the SORT1-mediated lysosomal degradation pathway. RPS23RG1 competed with SORT1 for MDGA2 binding to inhibit MDGA2 degradation. Furthermore, we showed that *Rps23rg1* knockout mice exhibited decreased MDGA2 levels and ASD-like behaviors, whereas restoration of MDGA2 levels attenuated social defects in* Rps23rg1* KO mice. Moreover, we identified a crucial region of RPS23RG1 for MDGA2 interaction and found that a peptide derived from this region not only bound MDGA2 and promoted MDGA2 levels, but also rescued social defects in *Mdga2*^+/-^ mice.

**Conclusion:** Our findings highlight a crucial role of RPS23RG1 in antagonizing SORT1-mediated lysosomal degradation of MDGA2 and suggest a potential for targeting the RPS23RG1-MDGA2 axis to treat ASD with MDGA2 deficiency.

## Introduction

Autism spectrum disorder (ASD) is a pediatric neurodevelopmental disorder characterized by social impairment, stereotyped and repetitive behaviors, delayed language development, and partial intellectual disability 1-3. Complex genetic alterations increase the risk of ASD 4,5. During the past decade, emerging studies have identified copy number variations (CNVs) and single nucleotide variations (SNVs) of multiple genes to be associated with ASD 6-9. A majority of these genes, such as *NRXNs*, *SHANKs*,* DLG4*, *NLGNs*, and *MDGA2*, encode synaptic proteins, indicating that synaptic dysfunction is one of the major causes of ASD 10-12.

MAM domain-containing glycosylphosphatidylinositol anchor 2 (MDGA2) belongs to the glycosylphosphatidylinositol (GPI)-dependent membrane-anchored protein family 13 and is known to regulate the trans-synaptic interaction between neurexins and neuroligins 14,15. Several genomic analyses have identified loss-of-function and missense mutations in the *MDGA2* gene in ASD cases 16-18. Furthermore, animal studies have shown that *Mdga2*^+/-^ mice with reduced MDGA2 expression exhibited increased repetitive behavior and social defects resembling those found in ASD patients 19,20, suggesting that MDGA2 deficiency plays a crucial role in the pathogenesis of ASD. Therefore, elucidating the regulatory mechanisms of MDGA2 may not only strengthen our understanding of ASD etiology but also identify new therapeutic targets.

RPS23RG1, a type IB transmembrane protein, is highly expressed in synapses 21. It has been shown in previous studies that the transmembrane domain of RPS23RG1 interacts with adenylate cyclases to regulate the adenylate cyclase/cAMP/protein kinase A/glycogen synthase kinase-3 signaling, and the intracellular domain of RPS23RG1 interacts with PSD-93/PSD-95 to maintain synaptic structure and function and interacts with p35 to restrain the CDK5/p35 kinase activity for tau phosphorylation 21-24. These findings suggest that RPS23RG1 may function as a scaffolding protein, interacting with other synaptic proteins to regulate their stabilities and functions. However, the specific interactions and regulatory mechanisms of the extracellular domain of RPS23RG1 with other proteins have yet to be elucidated.

In the present study, we found that the RPS23RG1 extracellular domain, especially the amino acid 103-128 region, interacted with MDGA2. Loss of RPS23RG1 resulted in decreased MDGA2 levels and ASD-like behaviors in mice, whereas restoration of MDGA2 rescued their social defects. Mechanistically, we showed that MDGA2 was subjected to SORT1-mediated lysosomal degradation and that RPS23RG1 inhibited MDGA2 degradation by competing with SORT1 for MDGA2 binding. Finally, we generated a peptide derived from RPS23RG1 and demonstrated that it not only bound MDGA2 to inhibit its degradation but also rescued social defects in *Mdga2*^+/-^ mice.

## Materials and Methods

### Mouse generation and genotyping

C57BL/6 wild-type (WT) mice were from Xiamen University Laboratory Animal Center. *Rps23rg1* knockout (KO, *Rps23rg1*^-/-^) mice in C57BL/6 background were reported previously 21. *Mdga2*^+/-^ mice (C57BL/6N background) with a deletion of exon 2 were purchased from Cyagen Biosciences. Mouse genotypes were determined by PCR, using mouse tail DNAs as templates and the following primer pairs:

*Rps23rg1*:

Forward: 5'-TTCGACGAAATCCAGCAACC-3'

Reverse: 5'-GTTCGTGCCCAATGATGGC-3'

*Mdga2*:

Forward-1: 5'-TCTCCATCTCCTGGTTGTCATTAG-3'

Forward-2: 5'-GAGAATACACTTCCGAGAAAGCTG-3'

Reverse: 5'-TGCTAATGGCTAGCAAATGCTG-3'

All mice were bred and maintained at Xiamen University Laboratory Animal Center. Male *Rps23rg1* KO mice, *Mdga2*^+/-^ mice, and WT mice aged 8-12 weeks were used for experiments. All animal procedures and protocols followed the guidelines of the National Institutes of Health Guide for the Care and Use of Laboratory Animals, and were approved by the Animal Ethics Committee of Xiamen University (XMULAC20200163, XMULAC20210167, and XMULAC20230232).

### Plasmids and siRNAs

Full-length human MDGA2 plasmid was generated using the pEGFP-N1 vector (YouBia) as a backbone. Full-length human RPS23RG1 and its truncated constructs (Δ57-67 and Δ103-128) were fused to the N-terminal Myc tag of the pCMV-Myc vector (Clontech). The extracellular domain of human RPS23RG1 was cloned into the pGEX-4T-1 (GE Healthcare).

FAM-labeled siRNAs were synthesized by GenePharma.

SORT1-siRNAs:

SiRNA-1 sense: 5'-GUCCUGAGAACUCUGGAAATT-3', antisense: 5'-UUUCCAGAGUUCUCAGGACTT-3;

SiRNA-2 sense: 5'-GCAGUAUGUUUGGCCAAAUTT-3', antisense: 5'-AUUUGGCCAAACAUACUGCTT-3;

SiRNA-3 sense: 5'-CCUCUGUGAUGGCUGAUAATT-3', antisense: 5'-UUAUCAGCCAUCACAGAGGTT-3';

SiRNA-4 sense: 5'-GCAUCAUUGUGGCCAUUGATT-3', antisense: 5'-UCAAUGGCCACAAUGAUGCTT-3'.

RPS23RG1-siRNAs:

SiRNA-1 sense: 5'-CCAGCAACCAGAGCUUUAUTT-3', antisense: 5'-AUAAAGCUCUGGUUGCUGGTT-3';

SiRNA-2 sense: 5'-CACUUCCUAAUGGCAGAAUTT-3', antisense: 5'-AUUCUGCCAUUAGGAAGUGTT-3';

SiRNA-3 sense: 5'-GAGACUCCUAGCAGUAUGATT-3', antisense: 5'-UCAUACUGCUAGGAGUCUCTT-3.

### Quantitative real-time reverse transcription PCR (qRT-PCR)

RNA was extracted and reverse-transcribed into cDNA. Gene expression was determined by qRT-qPCR using FastStart Universal SYBR Green Master Mix (ROX) (Roche). The primers used for qRT-PCR are as follows:

*Mdga2*: 5'-CCAGAGGCCTATGAAGTCCG-3' and 5'- TCCACAGTGAAATTCCCTCAA-3';

*RPS23RG1*: 5'-AGGGATGCAGAAGACAGGATTG-3' and 5'- AGTTCGCTTTCAGGTTGTCA-3';

β-actin: 5'-AGCCATGTACGTAGCCATCCA-3' and 5'-TCTCCGGAGTCCATCACAATG-3'.

### Cell culture and transfection

HEK293T, SH-SY5Y, and HeLa cells were obtained from ATCC and maintained in our laboratory. Cells were cultured in high-glucose DMEM (ThermoFisher) supplemented with 10% fetal bovine serum (ThermoFisher). Primary neurons were isolated from mouse embryos (E 16.5) and cultured in neurobasal medium (ThermoFisher) supplemented with 2% B27 (ThermoFisher) and 1 mM glutamine (ThermoFisher). The Turbofect transfection reagent (ThermoFisher) was used for plasmid transfection. The Lipofectamine RNAi Max (ThermoFisher) was used for siRNA transfection.

### Immunoblotting and co-immunoprecipitation (co-IP)

Equal protein amounts of total lysates from mouse brain tissues or cells were assayed by immunoblotting and co-IP as previously described 21. Protein lysates were immunoblotted with antibodies indicated, including: mouse anti-GAPDH (Abways, AB0038, 1:10,000), rabbit anti-β-actin (Cell Signaling Technology, 4967S, 1:1,000), mouse anti-Myc (Abmart, M20002L, 1:1,000), rabbit anti-GST (Proteintech, 10000-0-AP, 1:1,000), rabbit anti-MDGA2 (Abcam, ab135407, 1:1,000), mouse anti-GFP (Abmart, M20004L, 1:1,000), rabbit anti-GAD1 (Proteintech, 10408-1-AP, 1:1,000), rabbit anti-Gephyrin (Proteintech, 12681-1-AP, 1:1,000), rabbit anti-PSD-95 (Cell Signaling Technology, 3450S, 1:1,000), rabbit anti-SYN1 (Proteintech, 20258-1-AP, 1:1,000), rabbit anti-LAMP1 (Abcam, ab24170, 1:1,000), rabbit anti-ATP1A1 (Proteintech, 55187-1-AP, 1:1,000), rabbit anti-p35 (Cell Signaling Technology, 2680, 1:1,000), and rabbit anti-SORT1 (Proteintech, 12369-1-AP, 1:1,000). Mouse anti-mouse RPS23RG1 and rabbit anti-human RPS23RG1 antibodies were reported previously 21. The secondary antibodies used included Goat anti-Mouse IgG (H+L), HRP (ThermoFisher, 31430, 1:5,000) and Goat anti-Rabbit IgG (H+L), HRP (ThermoFisher, 31460, 1:5,000). For co-IP, 1 mg protein lysates were incubated with 1-2 μg antibodies or IgG, and 30 μl Protein G-Agarose beads (ThermoFisher, 20399) at 4°C overnight. Immunocomplexes were analyzed by immunoblotting. The intensity of the protein bands was quantified using the ImageJ software (National Institutes of Health).

### Immunofluorescence

Immunofluorescence was performed as previously described 21,24. Antibodies used were: mouse anti-Myc (Abmart, M20002L, 1:200), rabbit anti-MDGA2 (Abcam, ab135407, 1:200), rabbit anti-LAMP1 (Abcam, ab24170, 1:200), rabbit anti-SORT1 (Proteintech, 12369-1-AP, 1:200), rat anti-Gephyrin (Synaptic Systems, 147208, 1:200), mouse anti-PSD-95 (Millipore, MAB1596, 1:200), Alexa Fluor 635 Goat anti-Mouse IgG (H+L) (ThermoFisher, A-31575, 1:500), Alexa Fluor 488 Goat anti-Rabbit IgG (H+L) (ThermoFisher, A-11008, 1:500), and Alexa Fluor 594 Goat anti-Rat IgG (H+L) (ThermoFisher, A-11007, 1:500). Cell images were acquired by High Intelligent and Sensitive SIM (HIS-SIM, Guangzhou Computational Super-resolution Biotech). All specimens were imaged under identical conditions and analyzed using Image J software (National Institutes of Health).

### MDGA2 lentivirus and stereotaxic injection

Full-length human MDGA2 was packaged into lentiviruses (pCLenti-hSyn-EGFP-3xFLAG-P2A-HA-MDGA2) by OBiO Technology. MDGA2-expressing and control viruses were injected into the lateral ventricle of *Rps23rg1* KO and WT control male mice at postnatal day 7. Briefly, mice were anesthetized on ice for 2-3 minutes. Viruses were injected into the bilateral lateral ventricles using a 10-μL syringe (Hamilton) with a 30-G needle (Hamilton). After the virus injection, the pups were placed on a heating pad until they returned to normal activity. Subsequently, the pups were returned to the cage, covered with bedding, and ensured to be cared for and nursed by the mother.

### Peptide and intravenous injection

The following peptides were synthesized by Sangon Biotech, with conjugation of a biotin or fluorescein isothiocyanate (FITC) moiety bridged with a GGG linker at their amino termini: P1 (GRIWLFG), P2 (SYFSQHNSFCMRSTS), and scrambled peptide (FNSSFHRSTSMQSCY).

The peptides were dissolved in saline, with no more than 2% DMSO added to aid dissolution. 1-month-old *Mdga2*^+/-^ mice were administrated with P2 or scrambled control peptide via tail vein injection at 10 mg/kg/d for five consecutive days, followed by behavioral tests and biochemical analysis.

### *In vivo* imaging of the blood-brain barrier permeability of peptide

Cranial window preparation and multi-photon imaging were performed following a published protocol 25. Briefly, mice were anesthetized with isoflurane. A circular cranial window (4 mm in diameter) over the dura mater on the skull was created with a high-speed dental drill. A glass coverslip was then used to cover the cranial window and fixed on the skull. After recovering, mice were first injected with Texas Red Dextran (ThermoFisher, D1830) to visualize cortical vasculature and then injected with FITC-labeled peptide to evaluate its blood-brain barrier permeability, using FVMPE-RS multi-photon microscope (Olympus). Imaging data were collected using 830 nm and 920 nm laser excitations for Texas Red Dextran and FITC-labeled peptide, respectively.

### Lysosome isolation

The separation of lysosomes from the mouse brain was performed using the lysosome extraction kit (BestBio, BB-3603). Briefly, the brain was homogenized using a Dounce homogenizer. Lysosomal components were obtained through gradient centrifugation. The collected lysosomal precipitate was added to 1% TNEN lysate for protein extraction for subsequent experiments.

For lysosomal isolation from cells, we utilized a previously described lysosomal immunopurification method (LysoIP), using HeLa cells stably expressing 3xHA-tagged TMEM192 (HA-Lyso cells) 26. Briefly, treated HA-Lyso cells were rinsed twice with PBS and then scraped in KPBS (136 mM KCl, 10 mM KH_2_PO_4_, pH 7.25 adjusted with KOH). A portion of the cells was taken for total cell lysate preparation. The remaining cells were gently homogenized and centrifuged at 1,000 *g* for 2 min at 4°C. The supernatant containing organelles (including lysosomes) was incubated with KPBS-prewashed anti-HA beads (ThermoFisher, YF379158) on a gentle rotary shaker for 30 min. Immunoprecipitates were then gently washed with KPBS and resuspended in TNEN buffer for further study.

### Liquid chromatography-tandem mass spectrometry (LC-MS/MS)

The GST-fused RPS23RG1 extracellular fragment (RPS23RG1 aa 1-131) was expressed in bacteria and purified using glutathione Sepharose 4B (GE Healthcare, 17-0756-01). Mouse synaptosomes were prepared as previously described 21. Synaptosome proteins were dissolved in RIPA buffer and incubated with purified GST-RPS23RG1-ED protein. Precipitated proteins were subjected to proteomic analysis using LC-MS/MS with a Thermo Orbitrap Fusion Lumos instrument (ThermoFisher). Alternatively, GFP-tagged MDGA2 was transiently expressed in HEK293T cells. The membrane fraction was isolated from the total cell lysates using the Membrane and Cell Membrane Protein Extraction Kit (Phygene), and the membrane proteins were extracted and solubilized in 1% TNEN. MDGA2-interacting proteins were pulled down using an anti-GFP antibody and then subjected to LC-MS/MS for protein identification.

### Protein-protein interaction prediction

The PDB file of MDGA2 (AF-Q7Z553-F1) was from the SWISS-MODEL Protein Structure Database. The structure of RPS23RG1 (C-score=-4.62) was predicted by the I-TASSER platform (https://zhanggroup.org/I-TASSER, Zhang Lab). Protein-protein interactions were predicted from the GRAMM: Docking web server (http://gramm.compbio.ku.edu/gramm, Vakser Lab). Structural visualizations of different 3D protein models and their interactions were performed using PYMOL.

### Electrophysiological recordings

Miniature excitatory postsynaptic currents (mEPSCs) and miniature inhibitory postsynaptic currents (mIPSCs) were recorded from neurons in hippocampal slices of treated *Mdga2*^+/-^ mice and WT littermate controls in the presence of 1 μm TTX. The holding potentials for mEPSCs and mIPSCs were -70 mV and 0 mV, respectively. Internal solutions in pipettes contained 140 mM CsMeSO_3_, 5 mM TEA-Cl, 10 mM HEPES, 1 mM EGTA, 2.5 mM ATP magnesium salt, 0.3 mM GTP sodium salt, and 2 mM MgCl_2_-6H_2_O (pH 7.3, 298-300 mOsm).

### Behavioral tests

For the open field test, mice were placed in the center of the open field box, with the head of each mouse oriented in the same direction. Mice were allowed to explore freely for 10 min. Their total travel distance and time spent in the center of the box were recorded.

For the novel location recognition test, each mouse was first allowed to freely explore an open field box of 40 cm in length, width, and height for 10 min to get familiar with the box. On the second day, two identical objects, A and B, were placed in two diagonal positions in the open field box, and mice were allowed to explore objects A and B for 10 min freely. The time mice spent exploring each object was recorded. On the third day, object B was moved to another corner so that objects A and B were in parallel positions, and then mice were allowed to explore them freely for 10 min. The time mice spent exploring each object was recorded. The time mice spent exploring the new position of object B (T_B_) and the familiar position of object A (T_A_) was measured, and the recognition index [RI = T_B_/(T_B_ + T_A_)] was calculated for comparison.

For the nest-building test, a square cotton weighing 3 g was placed in a cage with fresh bedding. The test mouse was put into this cage and allowed to stay for 24 h (ensuring 12 h of light and 12 h of darkness). The nesting condition was then scored on a scale of 0 (cotton was intact and not even moved elsewhere), 1 (cotton was torn and placed haphazardly in various places in the cage), 2 (cotton was torn and gathered in one place in the cage, but loosely, with no apparent folding or molding), 3 (cotton was gathered and folded into a flat nest, with no obvious torn), 4 (cotton was gathered and folded into a three-dimensional nest with visible tearing but only one entrance and exit), or 5 (cotton was gathered and folded into a three-dimensional nest with visible tearing and two or more entrances and exits).

For the self-grooming test, each mouse was given 15 min to explore an open field box. The spontaneous behavior of each mouse was recorded and the self-groom behavior during the last 10 min was evaluated.

For the social affiliation test, a metal cage (8.5 cm diameter) containing a reference mouse (same gender as the experimental mice) was placed at a specific location in the open field (40 cm in diameter). Experimental mice were allowed to move freely in the open field for 10 min. Their exploration time in the field and sniffing time with the reference mouse were recorded.

For the three-chamber social interaction test, a rectangular, three-chambered box with one empty cage in each side chamber was used. Experimental mice were first allowed to habituate in the three chambers for 10 min. For the sociability test, a gender-matched strange mouse (S1) was placed in one empty cage, and experimental mice were allowed to explore freely for 8 min. The time spent sniffing S1 and the other empty cage (E) was recorded, and the sociability preference index (T(S1-E)/T(S1+E): time spent sniffing S1 minus time spent sniffing E over time spent sniffing both S1 and E) was calculated for comparison. For the social novelty test, another gender-matched strange mouse (S2) was placed into the empty cage, and experimental mice were allowed to explore freely for another 8 min. Their time spent sniffing S1 or S2 was measured, and the social novelty preference index (T(S2-S1)/T(S2+S1): time spent sniffing S2 minus time spent sniffing S1 over time spent sniffing both S2 and S1) was calculated for comparison.

### Statistical analysis

Statistical analysis was performed using GraphPad Prism 9. Data are presented as mean (Mean) ± standard error (SEM). Samples were first tested and found to accord with normal distribution. 2-tailed unpaired Student's t-test was then used for comparison between two independent samples, and one-way ANOVA with Tukey's multiple comparisons test was used for comparisons among multiple independent samples. The exact statistical method used for each comparison is provided in the corresponding figure legends. *P* < 0.05 is considered to be statistically significant.

## Results

### MDGA2 is a novel RPS23RG1-binding synaptic protein

**Figure 1:**
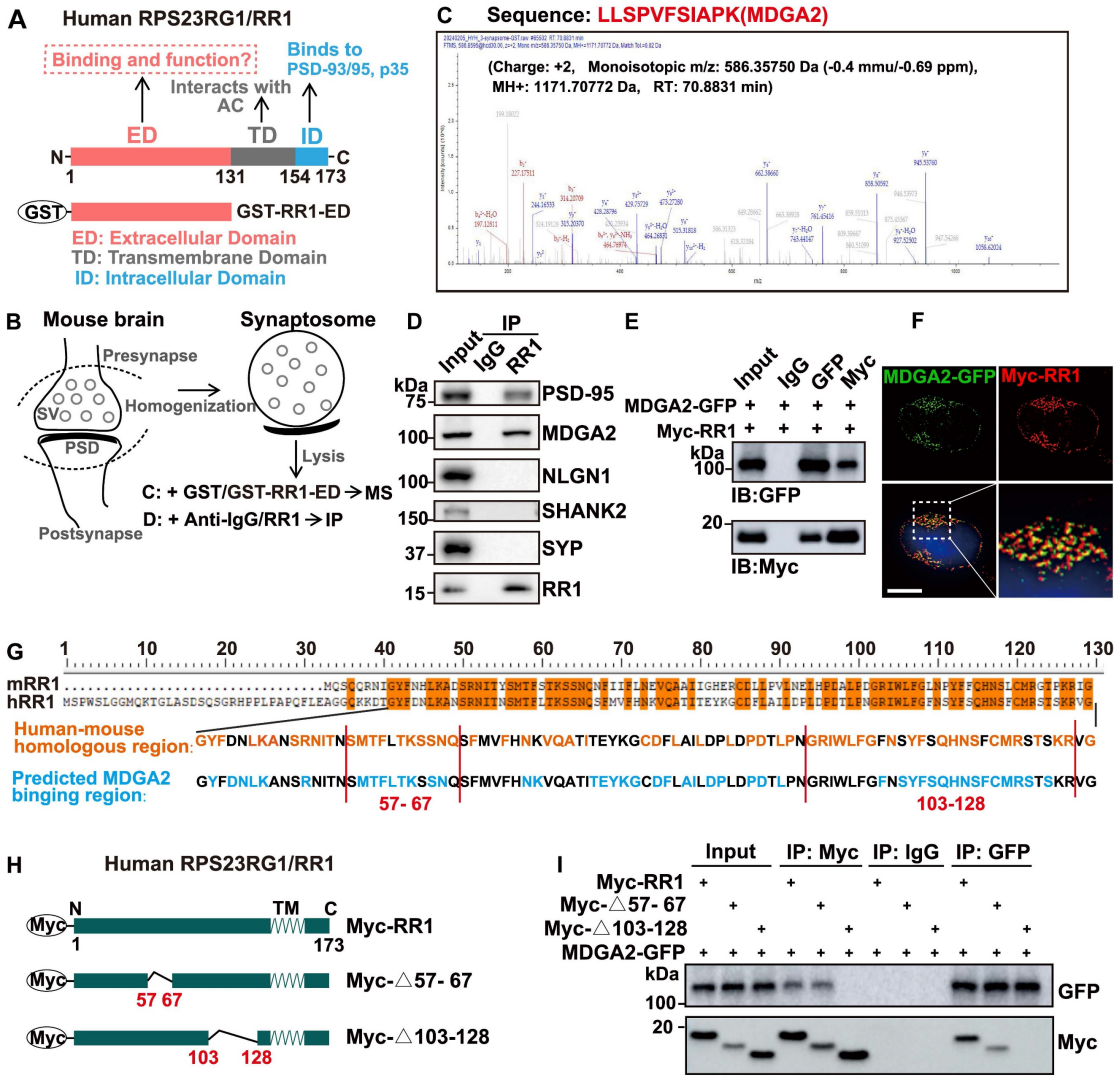
**MDGA2 is a novel RPS23RG1-binding synaptic protein. (A)** Schematic representation of the different structural domains and functions of human RPS23RG1 (RR1). **(B)** Schematic representation of synaptosome isolation and subsequent mass spectrometry (MS) or immunoprecipitation (IP) experiments. The extracellular domain of RPS23RG1 fused with GST (GST-RR1-ED) was incubated with synaptosome lysates, followed by pull-down using glutathione Sepharose 4B. Pulled-down proteins were subjected to MS for identification. Alternatively, pulled-down proteins were separated by SDS-PAGE and analyzed by immunoblotting. **(C)** Sequence of an MDGA2 peptide identified by MS analysis. **(D)** Protein lysates from synaptosomes were subjected to IP with IgG and an anti-RR1 antibody, and immunoblotting for the proteins indicated. **(E)** HEK293T cells were co-transfected with MDGA2-GFP and Myc-RPS23RG1 for 24 h. Protein lysates of these cells were subjected to IP with IgG and antibodies against GFP or Myc, and immunoblotting (IB) with antibodies against GFP or Myc. **(F)** HeLa cells co-transfected with MDGA2-GFP (green) and Myc-RPS23RG1 were immunostained with an anti-Myc antibody (red) and then counter-stained with DAPI (blue). The images were acquired using a High Sensitivity Structured Illumination Microscope (HIS-SIM). Scale bar: 10 μm. **(G)** Analysis of human RPS23RG1 (hRR1) and mouse RPS23RG1 (mRR1) extracellular domain sequences and predicted MDGA2-interacting sequences on hRR1. Orange represents identical amino acids between hRR1 and mRR1 proteins. Blue represents hRR1 protein residues predicted to interact with MDGA2 by PYMOL.** (H)** Schematic depictions of human RPS23RG1 and its truncated constructs. All constructs carry a Myc tag at the N-terminus. Δ57-67: lacking amino acids (aa) 57-67. Δ103-128: lacking aa 103-128. N: N-terminus. C: C-terminus. TM: transmembrane domain. **(I)** HEK293T cells were co-transfected with MDGA2-GFP and Myc-tagged full-length RR1, RR1-Δ57-67, or RR1-Δ103-128. Cell lysates were subjected to IP with IgG and antibodies against Myc or GFP, and immunoblotting with antibodies against GFP or Myc.

Previous studies have shown that the transmembrane domain (TD) of RPS23RG1 interacts with adenylate cyclases (AC) to regulate the downstream signaling and that the intracellular domain (ID) of RPS23RG1 interacts with PSD-93/PSD-95 to maintain synaptic structure and functional stability and interacts with p35 to restrain the CDK5/p35 kinase activity (Figure 1A). However, it is unknown whether the extracellular domain (ED) of RPS23RG1 interacts with other proteins and modulates their pathophysiological functions. To explore the potential binding of synaptic proteins by RPS23RG1 ED, we carried out affinity purification experiments using purified GST-RPS23RG1-ED protein to pull down interacting proteins from mouse brain synaptosome lysates, followed by mass spectrometry analysis (Figure 1A-B). The results showed that MDGA2 was one of the most abundant proteins identified (Figure 1C, Table S1). The interaction between MDGA2 and RPS23RG1 ED was confirmed through GST pull-down assays (Figure S1A). Moreover, we found that an anti-RPS23RG1 antibody co-immunoprecipitated endogenous MDGA2 and PSD-95 but not NLGN1, SHANK2, or SYP in mouse brain synaptosome lysates (Figure 1D). Exogenously expressed MDGA2 and RPS23RG1 also co-immunoprecipitated with each other in HEK293T cells (Figure 1E). In addition, exogenously-expressed RPS23RG1 and MDGA2 showed colocalization in HeLa cells (Figure 1F). These results demonstrate that MDGA2 binds to the extracellular domain of RPS23RG1.

We compared the extracellular domain sequences of human and mouse RPS23RG1 proteins and found that their sequence conservation was enriched in two regions: amino acids (aa) 57-67 and aa 103-128 (based on the human sequence, Figure 1G). A computer simulation prediction of the MDGA2-RPS23RG1 ED interaction also revealed that many RPS23RG1 ED amino acids possibly interacting with MDGA2 were located in the two regions (Figure 1G and Figure S1B). Therefore, we constructed truncated RPS23RG1 constructs lacking aa 57-67 (RR1-Δ57-67) or aa 103-128 (RR1-Δ103-128) (Figure 1H). When HEK293T cells were co-transfected with MDGA2 and the two RPS23RG1 truncated forms, we found that RR1-Δ57-67 but not RR1-Δ103-128 still bound MDGA2 (Figure 1I), suggesting that the region comprising aa 103-128 is crucial for the interaction between RPS23RG1 and MDGA2.

### Decreased MDGA2 mediates social deficits in *Rps23rg1* KO mice

Our previous study revealed that *Rps23rg1* KO mice had learning and memory deficits 21. Herein, we further found that although *Rps23rg1* KO mice showed no changes in locomotor activity (Figure S2C), they exhibited ASD-like behaviors, including increased self-grooming time resembling stereotyped repetitive behavior (Figure S2D), decreased nest-building score (Figure S2E), and decreased sociability preference (Figure S2F). Moreover, we found that MDGA2 protein levels (Figure S2A) but not their mRNA levels (Figure S2B) were dramatically decreased in the brain of *Rps23rg1* KO mice compared to WT controls.

**Figure 2:**
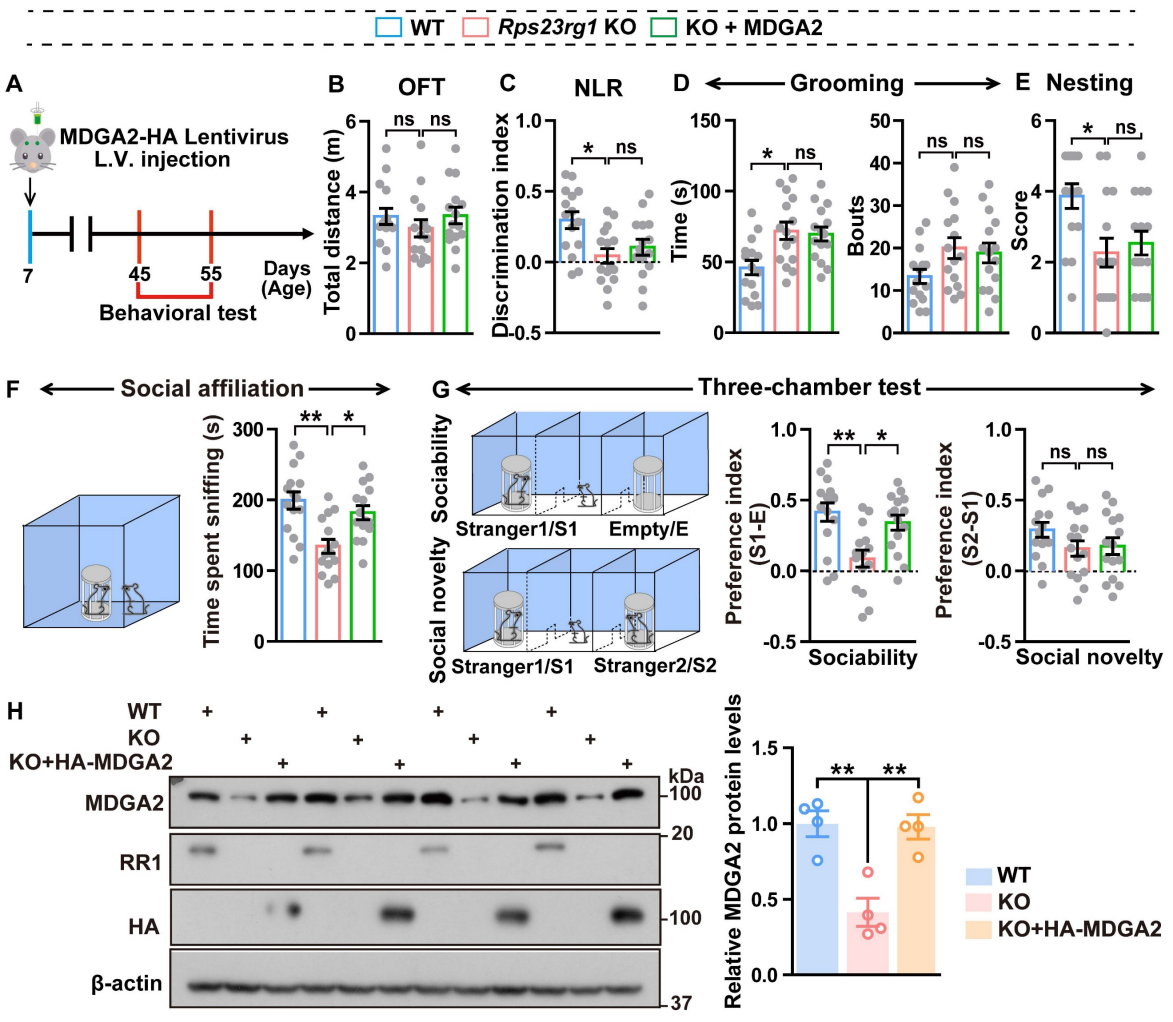
**Restoring MDGA2 attenuates social deficits in *Rps23rg1* KO mice. (A)** Schematic depiction of the experimental strategy. MDGA2-HA and control lentiviruses were bilaterally injected into the ventricles of P7 *Rps23rg1* KO mice. Control lentiviruses were also bilaterally injected into the ventricles of P7 wild-type (WT) mice. Mice were studied for their behaviors at 1.5-months of age. **(B-G)** Control WT, control *Rps23rg1* KO, and MDGA2-HA lentivirus-injected *Rps23rg1* KO mice were analyzed for their total travel distance in the open field test (OFT, **B**), their discrimination index in the novel location recognition test (NLR, **C**), their time spent self-grooming and bouts of self-grooming (**D**), their nesting score in the nest-building test (**E**), their time spent sniffing a reference mouse in the social affiliation test (**F**), and their sociability preference for sniffing a stranger mouse (S1) over an empty cylinder (E) and social novelty preference for sniffing a new stranger mouse (S2) over the familiar S1 mouse in the three-chamber test (**G**). n = 14 per group. **(H)** Equal protein amounts of cerebral lysates derived from studied mice were subjected to immunoblotting for the proteins indicated. MDGA2 protein levels were quantified by densitometry, normalized to those of β-actin, and compared to those of WT (set to one arbitrary unit). Data represent mean ± SEM. *P* values were determined by one-way ANOVA with Tukey's multiple comparisons test. *p < 0.05, **p < 0.01, ns: not significant.

Since loss-of-function mutations in MDGA2 are associated with ASD 16,18, we speculated that decreased MDGA2 protein levels might be responsible for ASD-like behaviors in *Rps23rg1* KO mice. Therefore, we delivered lentiviruses expressing MDGA2 into the bilateral ventricles of *Rps23rg1* KO mice at postnatal day 7 (P7) and studied their behaviors when mice were 1.5 months old (Figure 2A). We confirmed that the lentivirus injection normalized brain MDGA2 levels in *Rps23rg1* KO mice (Figure 2H). Although the restoration of MDGA2 had no impact on locomotor activity (Figure 2B), recognition memory (Figure 2C), repetitive self-grooming behavior (Figure 2D), nest-building ability (Figure 2E), or social novelty preference (Figure 2G), it significantly attenuated impaired social affiliation (Figure 2F) and sociability preference (Figure 2G) in *Rps23rg1* KO mice. These results suggest that MDGA2 deficiency directly contributes to the social impairments observed in *Rps23rg1* KO mice.

### RPS23RG1 regulates the lysosomal degradation of MDGA2

**Figure 3:**
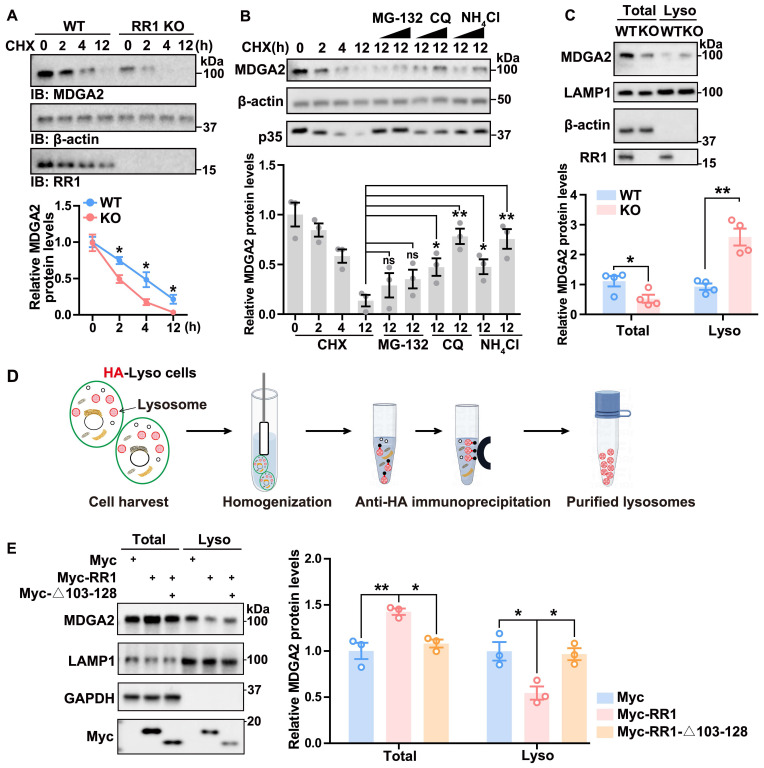
**RPS23RG1 regulates lysosomal degradation of MDGA2. (A)** Cultured primary neurons from WT and *Rps23rg1* KO mice were treated with 30 μM Cycloheximide (CHX) for the time indicated. MDGA2 levels were determined by immunoblotting, quantified by densitometry, and normalized to β-actin levels for comparison. The value at the 0 h time point was set to one arbitrary unit. n= 3 independent experiments.** (B)** HEK293T cells were exposed to 30 μM CHX along with varying concentrations of MG-132 (10 and 30 μM), CQ (30 and 100 μM), and NH_4_Cl (30 and 100 μM) for a duration of 12 h. MDGA2 levels were determined by immunoblotting, quantified by densitometry, and normalized to β-actin levels for comparison. The value at 0 h time point was set to one arbitrary unit. n = 3 independent experiments. P35 level change served as a positive control for the inhibition of proteasomal and lysosomal degradation. **(C)** Equal protein amounts of total lysates and lysosomal fraction lysates obtained from 1.5-month-old WT and *Rps23rg1* KO mouse brains were analyzed by immunoblotting for the proteins indicated. MDGA2 levels were quantified by densitometry, normalized to β-actin levels (for total) or LAMP1 levels (for lysosomal fraction), and compared to those of WT. n = 4 for total lysates, and n = 4 for lysosome fraction lysates. **(D)** Scheme of the workflow for the LysoIP method. HA-Lyso cells refer to HeLa cells stably expressing 3xHA-tagged TMEM192. **(E)** HA-Lyso cells were transfected with Myc-tagged full-length RR1, RR1-Δ103-128, and control vectors for 24 h. Equal protein amounts of total cell lysates and lysosomal fraction lysates were analyzed by immunoblotting for the proteins indicated. MDGA2 levels were quantified using densitometry, normalized to GAPDH (for total) or LAMP1 (for lysosomal fraction) levels, and compared to those of the control group (set to one arbitrary unit). n = 3. Data represent mean ± SEM. *P* values were determined by 2-tailed unpaired Student's t-test in (**A-C**) and one-way ANOVA with Tukey's multiple comparisons test in (**E**). *p < 0.05, **p < 0.01, ns: not significant.

Consistent with the MDGA2 reduction in *Rps23rg1* KO mice, we found that overexpression of RPS23RG1 led to increased MDGA2 protein levels (Figure S3A). Since *Mdga2* mRNA levels were not altered in *Rps23rg1* KO mice (Figure S2B), RPS23RG1 probably regulates MDGA2 at post-transcription levels. Indeed, when hippocampal neuronal cultures of *Rps23rg1* KO and WT mice were treated with cycloheximide (CHX) to inhibit protein synthesis, we found that MDGA2 degradation was significantly accelerated in *Rps23rg1* KO neurons compared to WT controls (Figure 3A), suggesting that RPS23RG1 regulates MDGA2 protein degradation. Furthermore, the degradation of MDGA2 was prevented by lysosomal inhibitors, including chloroquine (CQ) and NH_4_Cl, but not by the proteasome inhibitor MG-132 (Figure 3B). Moreover, although MDGA2 levels were less in total lysates of* Rps23rg1* KO mouse brain samples than those of controls, we found that they were much more in lysosomes isolated from *Rps23rg1* KO mouse brain samples than those from WT samples (Figure 3C). Together, these findings suggest that RPS23RG1 regulates the lysosomal localization and degradation of MDGA2.

To determine whether the RPS23RG1-MDGA2 interaction mediates the lysosomal degradation of MDGA2, we applied a lysosome immunoprecipitation (LysoIP) method, which uses an HA antibody to immunopurify lysosomes from HEK293T cells stabling expressing TMEM192 fused with the HA epitope (HA-Lyso cells, Figure 3D). When HA-Lyso cells were transfected with full-length RR1 or RR1-Δ103-128 that does not bind MDGA2, we found that overexpression of full-length RR1 led to an increase in MDGA2 levels in total cell lysates and a decrease in MDGA2 levels in lysosomal fraction lysates, whereas overexpression of RR1-Δ103-128 had no such effects (Figure 3E). Additionally, overexpression of full-length RR1 but not RR1-Δ103-128 significantly increased MDGA2 levels on the cell surface (Figure S3B).

### RPS23RG1 competes with SORT1 for MDGA2 binding and suppresses the lysosomal degradation of MDGA2

**Figure 4:**
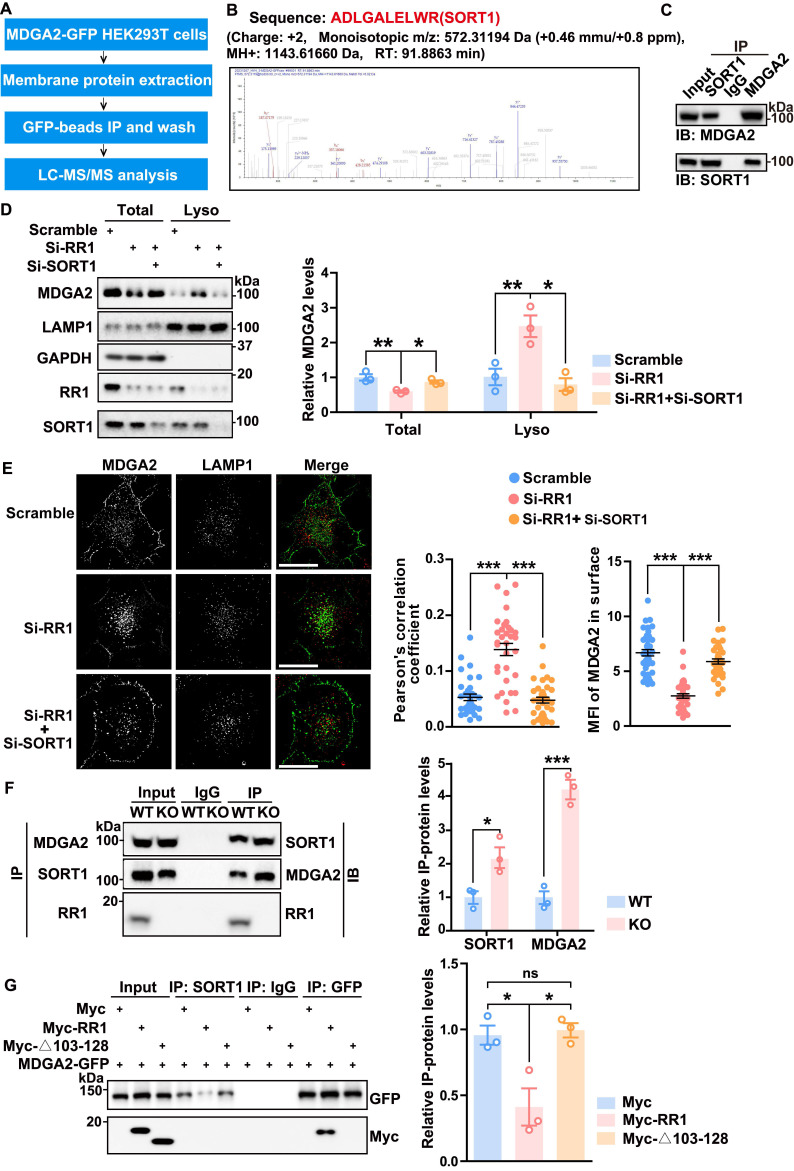
**RPS23RG1 competes with SORT1 for MDGA2 binding and suppresses the lysosomal degradation of MDGA2. (A)** Schematic representation of the strategy for identifying MDGA2-interacting proteins. **(B)** Sequence of a SORT1 peptide identified by LC-MS analysis. **(C)** Protein lysates from a 1.5-month-old WT mouse brain were subjected to immunoprecipitation (IP) with IgG and antibodies against SORT1 or MDGA2 and immunoblotting (IB) with antibodies against MDGA2 or SORT1. **(D)** HA-Lyso cells were transfected with Scramble, Si-RR1 (RPS23RG1), and Si-RR1 plus Si-SORT1 for 48 h. Equal protein amounts of total cell lysates and lysosomal fraction lysates were analyzed by IB for the proteins indicated. MDGA2 levels were quantified by densitometry, normalized to GAPDH (for total) or LAMP1 (for lysosomal fraction) levels, and compared to those of the control group (set to one arbitrary unit). n = 3. **(E)** HeLa cells were first transfected with MDGA2-GFP (green) and then transfected with Scramble, Si-RR1, and Si-RR1 plus Si-SORT1 for 48 h. Subsequently, cells were immunostained with an anti-LAMP1 antibody (red). Images were captured using a High Sensitivity Structured Illumination Microscope (HIS-SIM). Scale bars: 10 μm. The levels of MDGA2 in lysosomes (colocalized with LAMP1) and on the cell surface were quantified using Image J and compared to those of the control group. n = 40 cells from three independent experiments per group. **(F)** Equal amounts of protein lysates from 1.5-month-old WT and *Rps23rg1* KO mouse brains were subjected to IP with anti-MDGA2 and anti-SORT1 antibodies and then IB for the proteins indicated. Protein levels were quantified by densitometry, normalized to input levels, and compared to their respective controls (set to one arbitrary unit). n = 3. **(G)** HEK293T cells were co-transfected with MDGA2-GFP and Myc-tagged full-length RR1 or Δ103-128. Cell lysates were subjected to IP with antibodies against GFP or SORT1 and IB with antibodies against GFP or Myc. Protein levels were quantified by densitometry, normalized to input levels, and compared to controls (set to one arbitrary unit). n = 3 independent experiments. Data represent mean ± SEM. *P* values were determined by 2-tailed unpaired Student's t-test in (**F**) and one-way ANOVA with Tukey's multiple comparisons test in (**D, E, G**). *p < 0.05, **p < 0.01, ***p < 0.001, ns: not significant.

MDGA2 belongs to a group of GPI-anchored membrane proteins primarily degraded through transcytotic endocytosis mediated by binding proteins 13. To identify proteins that directly control MDGA2 degradation, we overexpressed MDGA2-GFP in HEK293T cells. Membrane proteins were then extracted and immunoprecipitated with GFP-beads, and pulled-down proteins were subjected to LC-MS/MS analysis (Figure 4A). A SORT1 peptide was identified through this process (Figure 4B, Table S2). Co-immunoprecipitation assays confirmed the interaction between exogenous MDGA2 and endogenous SORT1 (Figure S4A). Immunofluorescence staining further showed the colocalization between exogenous MDGA2 and endogenous SORT1 both on the cell surface (non-permeabilized) and within the cell (permeabilized) (Figure S4B). Moreover, anti-MDGA2 and anti-SORT1 antibodies co-immunoprecipitated SORT1 and MDGA2, respectively, in mouse brain samples (Figure 4C), demonstrating the interaction between endogenous MDGA2 and endogenous SORT1.

A previous study reported the involvement of SORT1 in the lysosomal degradation of PrPC, a GPI-anchored glycoprotein located at the cell surface 27. To investigate whether SORT1 also regulates the lysosomal degradation of MDGA2, we designed four SORT1-targeting siRNAs and found that all of them significantly downregulated SORT1 (Figure S4C). We selected the most efficient SORT1 siRNA (Si-4, named Si-SORT1 thereafter) for the following experiments and found that SORT1 knockdown in HEK293T cells resulted in an upregulation of MDGA2 protein levels (Figure S4D).

We also designed three RPS23RG1-targeting siRNAs and assessed their knockdown efficiency. Si-RR1(1) could significantly downregulate RPS23RG1 expression and was used for the following experiments (Figures S5A-B). As expected, RPS23RG1 knockdown in HA-Lyso cells decreased and increased MDGA2 levels in total cell lysates and lysosomal fraction lysates, respectively (Figure 4D). While additional SORT1 knockdown reversed MDGA2 level changes in these lysates (Figure 4D). Consistently, immunofluorescence staining revealed that the increased lysosomal localization and the decreased cell surface localization of MDGA2 upon RPS23RG1 knockdown were reversed by additional SORT1 knockdown (Figure 4E). These findings highlight the role of SORT1 as a novel MDGA2-binding protein in regulating the lysosomal degradation of MDGA2.

We next studied and found that SORT1 levels remained unaltered in the cortex and hippocampus of *Rps23rg1* KO mice compared to those of WT littermates (Figure S5C). Co-immunoprecipitation experiments showed no interaction between SORT1 and RPS23RG1 (Figure S5D). However, an anti-MDGA2 antibody co-immunoprecipitated more SORT1, and an anti-SORT1 antibody co-immunoprecipitated more MDGA2 in *Rps23rg1* KO mouse brain samples compared to controls (Figure 4F), suggesting an enhanced interaction between MDGA2 and SORT1 upon RPS23RG1 deficiency. In cells co-transfected with MDGA2 and full-length RPS23RG1 or RPS23RG1-Δ103-128, an anti-SORT1 antibody co-immunoprecipitated less MDGA2 in the presence of full-length RPS23RG1 but not in the presence of RPS23RG1-Δ103-128 (Figure 4G), suggesting that the binding of RPS23RG1 to MDGA2 inhibits the interaction between MDGA2 and SORT1. These findings indicate that RPS23RG1 competes with SORT1 for binding to MDGA2 through the 103-128 fragment.

### RPS23RG1-derived peptide inhibits MDGA2 lysosomal degradation

**Figure 5:**
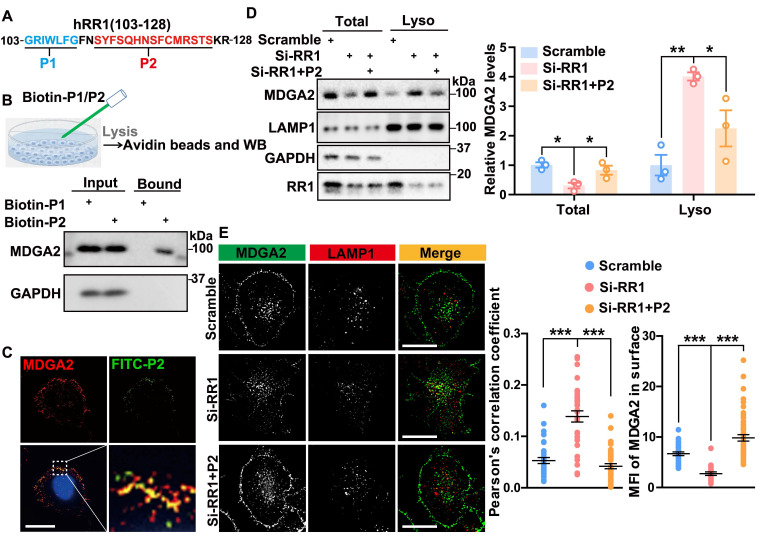
**RPS23RG1-derived peptide inhibits lysosomal degradation of MDGA2. (A)** Scheme of P1 (aa 103-109) and P2 (aa 112-126) peptides derived from the RR1 103-128 region. **(B)** HeLa cells were incubated with biotin-labeled P1 or P2 peptide (100 nM) for 16 h. Cell lysates were incubated with avidin beads, and precipitated proteins were immunoblotted with an antibody against MDGA2.** (C)** HeLa cells were incubated with FITC-labeled P2 peptide (100 nM, green) for 16 h, and then immunostained with an antibody against MDGA2 (red) and stained with DAPI (blue). Images were acquired by High Sensitivity Structured Illumination Microscope (HIS-SIM). Scale bar: 10 μm. **(D)** HA-Lyso cells were transfected with Scramble and Si-RR1 and incubated with or without P2 peptide for 48 h. Equal protein amounts of total cell lysates and lysosome fraction lysates were analyzed by immunoblotting for the proteins indicated. MDGA2 levels were quantified by densitometry, normalized to GAPDH (for total) or LAMP1 (for lysosomal fraction) levels, and compared to those of the control group (set to one arbitrary unit). n = 3 independent experiments. **(E)** HeLa cells were first transfected with MDGA2-GFP (green), then transfected with Scramble and Si-RR1, and incubated with or without P2 peptide for 48 h. Cells were immunostained with an antibody against LAMP1 (red). Images were acquired by High HIS-SIM. Scale bars: 10 μm. MDGA2 levels in lysosomes (colocalized with LAMP1) and on the cell surface were quantified by Image J and compared to those of controls. n = 40 cells from 3 independent experiments per group. Data represent mean ± SEM. *P* values were determined by one-way ANOVA with Tukey's multiple comparisons test. *p < 0.05, **p < 0.01, ***p < 0.001.

To further determine the interaction sites between RPS23RG1 and MDGA2, we selected two relatively conserved segments, P1 (aa 103-109) and P2 (aa 112-126) within the RPS23RG1 103-128 fragment (Figure 5A). Biotin pull-down assays were conducted by incubating the biotin-conjugated P1 and P2 peptides with cells. The results showed that P2 but not P1 pulled down MDGA2 (Figure 5B). Immunofluorescence staining of cells treated with FITC-labeled P2 also showed marked colocalization between MDGA2 and P2 peptide on the cell surface (Figure 5C). We further studied whether P2 can prevent MDGA2 degradation. When HEK293T cells subjected to RPS23RG1 knockdown were treated with P2, we found that the decreased MDGA2 levels in total cell lysates and the increased MDGA2 levels in lysosomal fraction lysates upon RPS23RG1 knockdown were reversed (Figure 5D). Immunofluorescence staining also confirmed that P2 treatment reversed the increased lysosomal localization and the decreased cell surface localization of MDGA2 in RPS23RG1 knockdown cells (Figure 5E).

### RPS23RG1-derived peptide attenuates social impairments in MDGA2-deficient mice

MDGA2-deficient mice exhibit ASD-like behaviors 19,20. Considering that the P2 peptide can restore MDGA2 levels in cells, we studied whether P2 can alleviate ASD-like behaviors in MDGA2-deficient mice. When FITC-labeled P2 peptide was injected into *Mdga2*^+/-^ mice through tail vein administration, we observed that it crossed the cortical vasculature and diffused in the parenchyma 90 s after injection (Figure S6A). We also observed both FITC-labeled P2 and a scrambled peptide in cortical and hippocampal regions 12 h after they were intravenously injected in mice (Figure S6B). These results indicate that these peptides can pass the blood-brain barrier (BBB).

**Figure 6:**
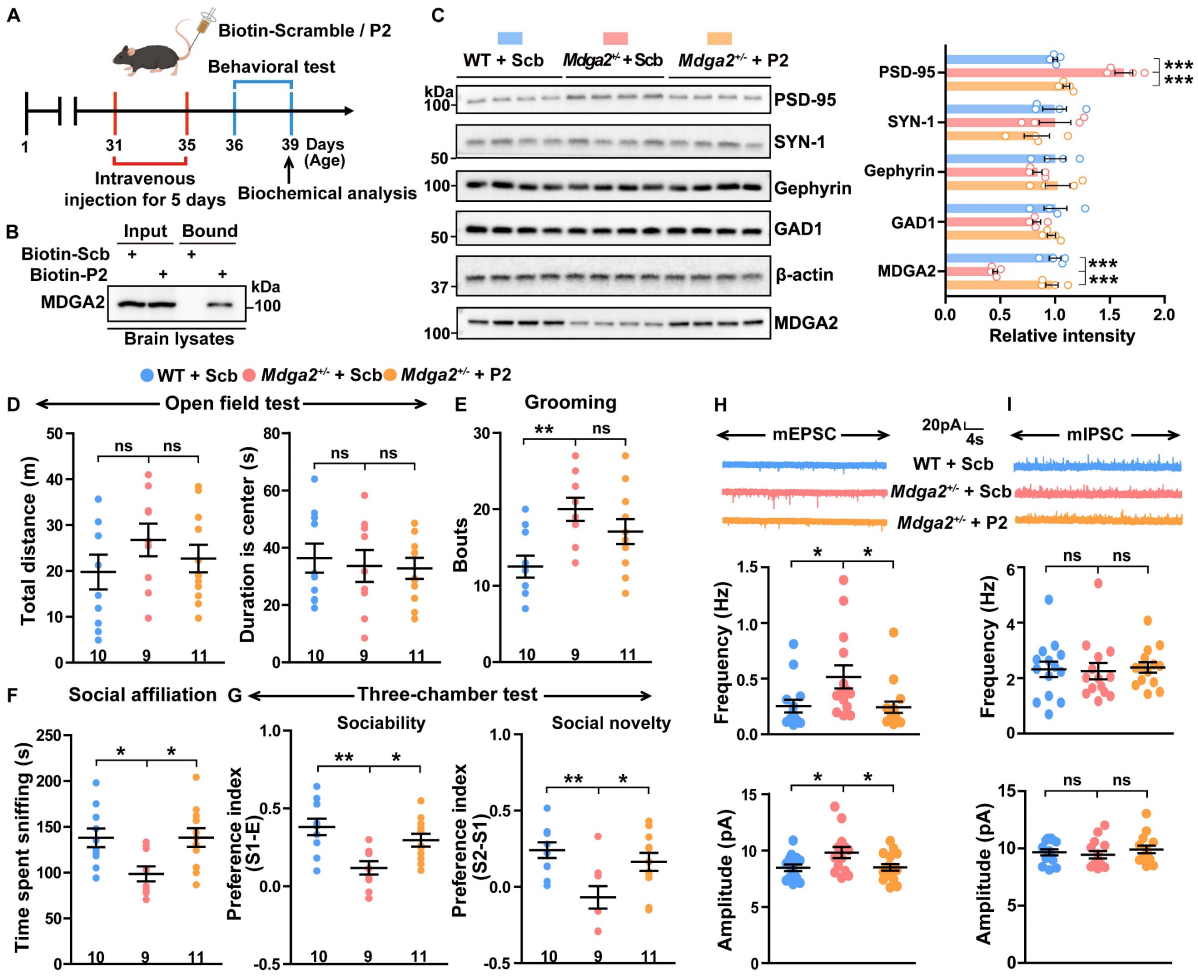
**RPS23RG1-derived peptide attenuates social deficits in MDGA2-deficient mice. (A)** Schematic depiction of the experimental strategy. One-month-old *Mdga2*^+/-^ mice and their WT littermate controls were administered with 10 mg/kg weight scrambled (Scb) or P2 peptide via intravenous injection once daily for 5 days. Mice were then subjected to behavioral tests and biochemical analysis. **(B)** Mice were sacrificed after a 5-day intravenous injection of biotin-labeled peptides. Equal protein amounts of brain lysates were precipitated with avidin beads and then immunoblotted with an antibody against MDGA2. **(C)** Brain tissues were taken from treated mice. Equal protein amounts of brain lysates were immunoblotted for the proteins indicated. Protein levels were quantified by densitometry, normalized to β-actin levels, and compared to WT controls (set to one arbitrary unit). n=4 per group. **(D-G)** Treated mice were analyzed for their total travel distance and duration in the center in the open field test (**D**), their bouts of self-grooming (**E**), their sniffing time in the social affiliation test (**F**), and their sociability and social novelty preferences in the three-chamber test (**G**). WT + Scb, n =10; *Mdga2*^+/-^ + Scb, n = 9; *Mdga2*^+/-^ + P2, n = 11. **(H, I)** The mEPSCs (**H**) and mIPSCs (**I**) of hippocampal neurons in treated mice were recorded, and their frequencies and amplitudes were quantified for comparison. n = 15 cells from 3 mice per group for mEPSCs; n = 14 cells from 3 mice per group for mIPSCs. Data represent mean ± SEM. *P* values were determined by one-way ANOVA with Tukey's multiple comparisons test. *p < 0.05, **p < 0.01, ***p < 0.001, ns: not significant.

We then carried out intravenous administration of biotin-labeled P2 and control peptide into *Mdga2*^+/-^ mice for 5 d, followed by behavioral and biochemical assessments (Figure 6A). Intravenously administered biotin-P2 was found to pull down MDGA2 in brain tissues (Figure 6B), further indicating its ability to cross BBB and target MDGA2 in the brain. Furthermore, P2 treatment restored the decreased MDGA2 levels in* Mdga2*^+/-^ mouse brain to be comparable to those in WT controls (Figure 6C). Importantly, treatment with P2 significantly rescued social affiliation deficits during the social affiliation test (Figure 6F) and impaired sociability and social novelty preferences during the three-chamber test (Figure 6G) in *Mdga2*^+/-^ mice. However, P2 treatment did not affect mouse locomotor activity in the open field test (Figure 6D) and reverse increased repetitive self-grooming behavior in *Mdga2*^+/-^ mice (Figure 6E). Together, these results indicate that P2 treatment can attenuate social impairments associated with MDGA2 deficiency.

We also studied several synaptic proteins and found that the levels of PSD-95 but not those of Gephyrin, SYN-1, or GAD1 were significantly decreased in *Mdga2*^+/-^ mice compared to controls. Meanwhile, P2 peptide treatment reversed the reduction of PSD-95 without affecting other synaptic proteins (Figure 6C). Additionally, we conducted whole-cell recordings of CA1 pyramidal neurons in hippocampal slices from treated mice. The results showed that both the frequency and amplitude of mEPSCs increased in *Mdga2*^+/-^ mice, and such increases were reversed by P2 peptide treatment (Figure 6H). However, no differences in the frequency and amplitude of mIPSCs were observed between *Mdga2*^+/-^ mice and controls, nor did P2 peptide treatment affect mIPSC frequency or amplitude in *Mdga2*^+/-^ mice (Figure 6I). Finally, we found that MDGA2 colocalized with both PSD-95 and Gephyrin in cultured hippocampal neurons (Figure S7).

## Discussion

RPS23RG1 is a novel type IB protein. RPS23RG1 deficiency plays a crucial role in Alzheimer's disease, and *Rps23rg1* KO mice exhibit dramatic learning and memory deficits 21,24. Previous studies have shown that the RPS23RG1 transmembrane domain interacts with adenylyl cyclases to modulate the PKA/GSK-3 signaling pathway 23, and its intracellular domain interacts with PSD-93/PSD-95 to maintain normal synaptic function and with p35 to inhibit the CDK5/p35 kinase activity 21,24. In the present study, we further found that the extracellular domain of RPS23RG1 interacted with MDGA2.

Several genome-wide analyses have identified loss-of-function and missense mutations in *MDGA2* associated with ASD 16-18. MDGA2 haploinsufficiency results in ASD-like behaviors in mice, whereas complete loss of MDGA2 leads to embryonic lethality 19,28. Herein, we found that *Rps23rg1* KO mice had decreased MDGA2 levels and exhibited ASD-like behaviors. Restoring MDGA2 levels in *Rps23rg1* KO mice reversed their social defects, suggesting that RPS23RG1 modulates social behaviors by maintaining MDGA2 levels.

MDGA2 is a synaptic protein anchored to the plasma membrane through a GPI-dependent mechanism and participates in synaptic functions. However, the predominant expression of MDGA2 in either excitatory or inhibitory neurons and its function in these two types of neurons remain a topic of debate. Early studies suggested that MGDA2 primarily interacted with NLGN2 and interfered with inhibitory synapse development without affecting NLGN1 14,15. However, later studies using MDGA2-deficient mice found that MDGA2 preferentially interacted with NLGN1 and negatively regulated the NLGN1-NRXN synaptic pathway to suppress excitatory synapse development, and MDGA2 deficiency led to elevated excitatory synaptic activity in mice 19,28. Other studies also found that MDGA2 deficiency enhanced excitatory synapse functions 29,30. Another study showed that MDGA2 deficiency had no effect on the abnormal cytosolic Gephyrin aggregation, the reduction in inhibitory synaptic transmission, and the exacerbated anxiety-related behavior in *Nlgn2* knockout mice 31. Here we also found that MDGA2 deficiency resulted in elevated excitatory synaptic activity and PSD-95 levels, without affecting inhibitory synaptic activity and inhibitory synapse-related proteins. Together, these findings suggest that MDGA2 is expressed in both excitatory and inhibitory neurons but exerts functions preferentially in excitatory neurons.

Mechanistically, we found that RPS23RG1 constrained the lysosomal degradation of MDGA2 by competing with SORT1 for MDGA2 binding. SORT1 is a VPS10p domain sorting protein primarily localized in the trans-Golgi network (TGN). SORT1 is widely expressed in neurons and crucial for cargo transport between the TGN and late endosomes 32-35. Some studies have shown that SORT1 can interact with GPI proteins, such as PrPc, and guide them toward the lysosomal pathway for degradation 27. We revealed that MDGA2 was also degraded through the SORT1-mediated lysosomal degradation pathway. More importantly, we found that RPS23RG1 deficiency resulted in an increased interaction between SORT1 and MDGA2, whereas SORT1 knockdown reversed the elevated lysosomal degradation of MDGA2 upon RPS23RG1 deficiency.

Decreased MDGA2 levels in mice result in ASD-like phenotypes, suggesting that restoring MDGA2 levels by inhibiting its lysosomal degradation may become a strategy for ASD intervention. We identified the core RPS23RG1 region for MDGA2 binding and showed that a peptide derived from this core RPS23RG1 region not only restored MDGA2 levels but also attenuated social defects in MDGA2-deficient mice.

We found that AAV-mediated expression of MDGA2 only attenuated social affiliation and sociability deficits but not other ASD-like behaviors in *Rps23rg1* KO mice, and that P2 peptide treatment only attenuated social affiliation, sociability, and social novelty deficits, without affecting other ASD-like behaviors in *Mdga2*^+/-^ mice. At this time, we cannot explain why restoring MDGA2 levels only attenuated social deficits but not other ASD-like behaviors such as increased repetitive behavior in the two types of mice. Since social and repetitive behaviors have both common and unique neural circuits 36-38, one possibility is that AAV-mediated MDGA2 expression and P2 peptide treatment might affect the neural circuits specifically associated with social behaviors more profoundly than those specifically associated with repetitive behavior. Alternatively, neural circuits and/or synapses responsible for social behaviors are less impaired than those responsible for repetitive behavior by RPS23RG1 and MDGA2 deficiencies, making them more resilient to MDGA2 restoration. In some other studies, it was also found that certain treatments, such as citalopram and Akt and mTOR inhibitors, specifically rescued social deficits without affecting repetitive behavior in some ASD model mice 39,40. Nevertheless, further studies are required to elucidate the underlying mechanism.

Taken together, we show that the synaptic membrane protein RPS23RG1 plays a protective role in ASD by interacting with MDGA2 and constraining its lysosomal degradation. We also reveal, for the first time, that MDGA2 degradation primarily occurs through the SORT1-mediated lysosomal pathway. Notably, we demonstrate that inhibiting MDGA2 degradation using a peptide derived from RPS23RG1 attenuates social defects in MDGA2-deficient mice. These findings identify a protective role of RPS23RG1 in ASD and offer novel approaches to targeting RPS23RG1 and MDGA2 for disease intervention.

## Supplementary Material

Supplementary figures.

Supplementary table 1.

Supplementary table 2.
