# Supplementary material for: RPS23RG1 inhibits SORT1-mediated lysosomal degradation of MDGA2 to protect against autism
Source: Theranostics. 2025 Jan 1;15(4):1338–52. doi: 10.7150/thno.100451 (PMC11729561; doi:10.7150/thno.100451)
Supplement: Supplementary file 1 — Supplementary figures. [file thnov15p1338s1.pdf]

Supplemental information

RPS23RG1 inhibits SORT1-mediated lysosomal degradation of MDGA2 to protect against autism

Yuanhui Huo et al.

Figures S1-7

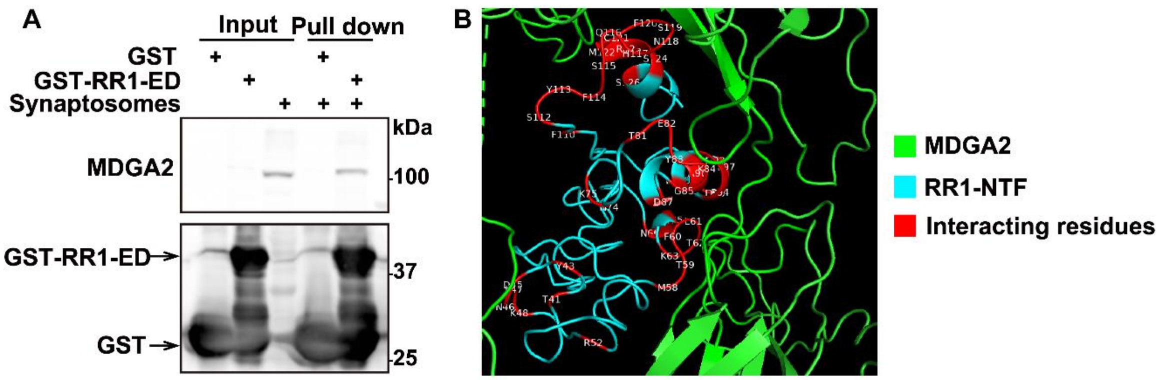

Figure S1. The extracellular domain of RPS23RG1 interacts with MDGA2. (A) GST-fused extracellular domain of RPS23RG1 (GST-RR1-ED) and control GST proteins were incubated with synaptosome lysates and Glutathione Sepharose 4B. Pulled-down proteins were subjected to immunoblotting. **(B)** Structure prediction of the RPS23RG1-ED and MDGA2 interaction by PYMOL with Interface Residues.

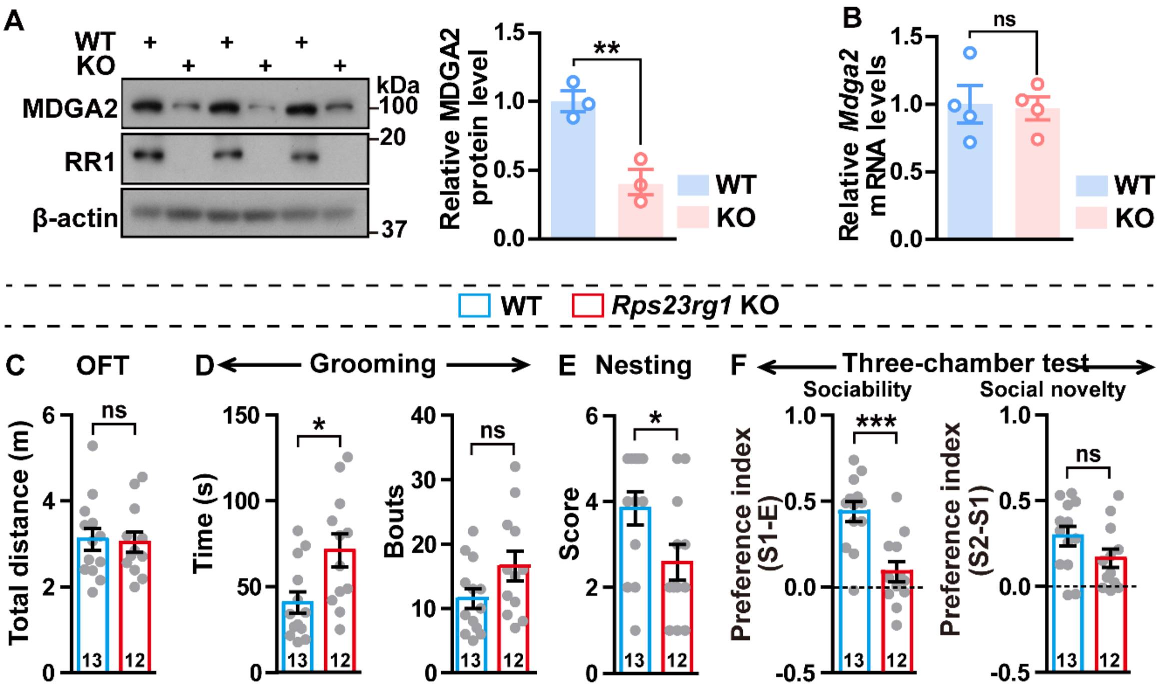

Figure S2. *Rps23rg1* KO mice exhibit decreased MDGA2 levels and ASD-like behaviors. (A) Equal protein amounts of cerebral samples derived from 1.5-month-old WT and *Rps23rg1* KO mice were immunoblotted for MDGA2, RPS23RG1 (RR1), and β-actin. MDGA2 protein levels were quantified by densitometry, normalized to β-actin levels, and compared to those of WT (set to one arbitrary unit). n = 3 per group. **(B)** *Mdga2* mRNA levels in the brains of WT and *Rps23rg1* KO mice were analyzed by qRT-PCR, normalized to those of β-actin, and compared to those of WT (set to one arbitrary unit). n = 4. **(C-F)** 1.5-month-old WT and *Rps23rg1* KO mice were analyzed for their total travel distance in the open field test (**C**), their time spent self-grooming and bouts of self-grooming (**D**), their nesting score in the nest-building test (**E**), and their sociability preference for sniffing a stranger mouse (S1) over an empty cylinder (E) and social novelty preference for sniffing

a new stranger mouse (S2) over the familiar S1 mouse in the three-chamber test (**F**). WT: n = 13; KO: n = 12. Data represent mean ± SEM. *P* values were determined by 2-tailed unpaired Student's t-test. *$p < 0.05$, **$p < 0.01$, ***$p < 0.001$, ns: not significant.

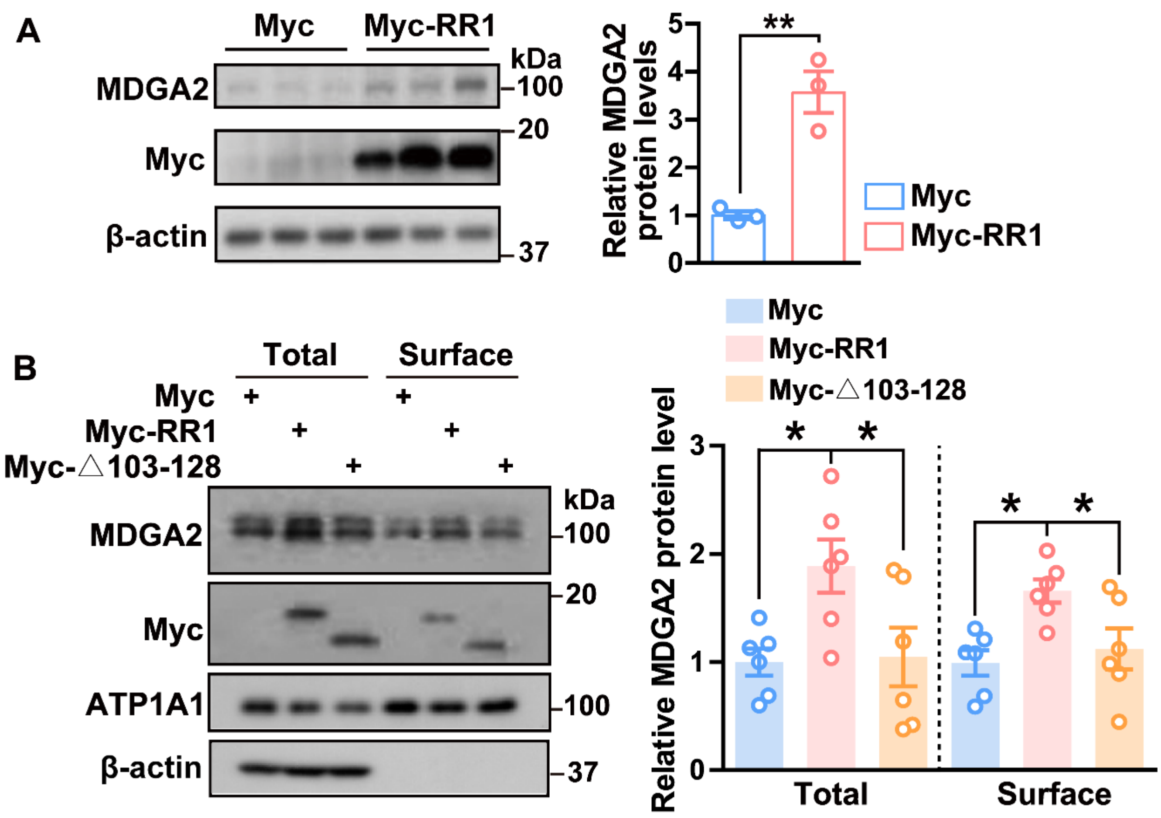

Figure S3. RPS23RG1 overexpression promotes MDGA2 protein levels. (A) HEK293T cells were transfected with Myc-RPS23RG1 (RR1) and Myc control vectors for 24 h. Equal protein amounts of cell lysates were immunoblotted for the proteins indicated. MDGA2 protein levels were quantified by densitometry, normalized to β-actin levels, and compared to those of control (set to one arbitrary unit). n = 3. **(B)** HEK293T cells were transfected with Myc-tagged full-length RPS23RG1, RPS23RG1-Δ103-128, and Myc control vectors for 24 h. Cell surface proteins were precipitated by surface biotinylation assays. Total cell lysate proteins and cell surface proteins were immunoblotted for the proteins indicated. MDGA2 protein levels were quantified by densitometry, normalized to β-actin (for total) and ATP1A1 (for cell surface) levels, respectively, and compared to

respective controls (set to one arbitrary unit). n = 6. Data represent mean ± SEM. *P* values were determined by 2-tailed unpaired Student's t-test in (**A**) and one-way ANOVA with Tukey's multiple comparisons test in (**B**). $^*p < 0.05$, $^{**}p < 0.01$.

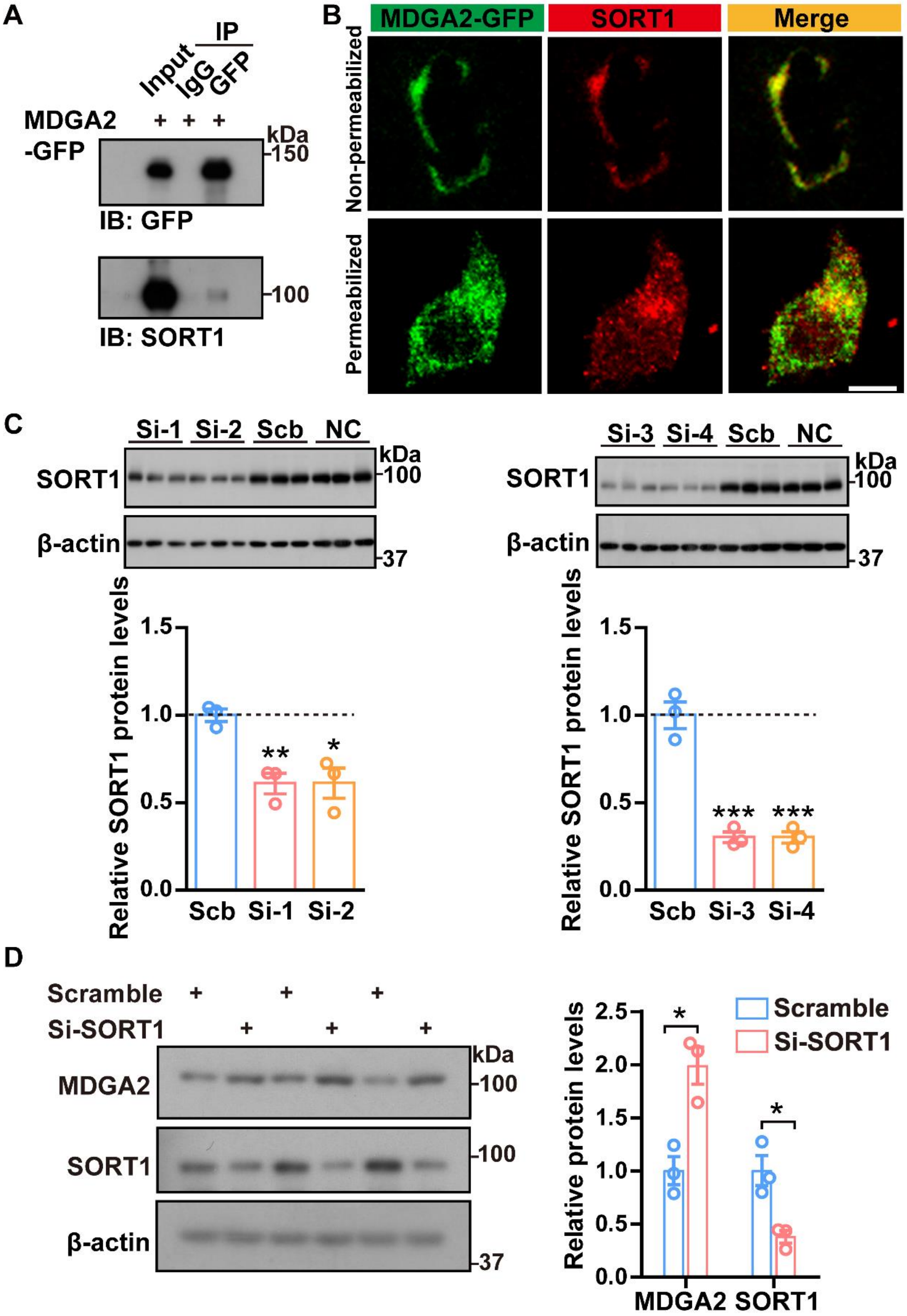

Figure S4. SORT1 interacts with MDGA2 and regulates MDGA2 levels. (A)

HEK293T cells were transfected with MDGA2-GFP. Equal protein amounts of cell lysates were subjected to immunoprecipitation (IP) with IgG and an anti-GFP antibody, followed by immunoblotting (IB) with antibodies against GFP or SORT1. **(B)** HeLa cells were transfected with MDGA2-GFP (green). Permeabilized and non-permeabilized cells were immunostained with an antibody against SORT1 (red) and observed under a confocal microscope. Scale bar: 10 μm. **(C)** HEK293T cells were transfected with Scramble (Scb) and different SORT1 siRNAs (Si-1, Si-2, Si-3, and Si-4) for 48 h. Equal protein amounts of lysates from treated and untreated (NC) cells were immunoblotted for the proteins indicated. SORT1 protein levels were quantified by densitometry, normalized to β-actin levels, and compared to those of Scb (set to one arbitrary unit). n=3. **(D)** HEK293T cells were transfected with Scramble and si-SORT1 for 48 h. Equal protein amounts of cell lysates were immunoblotted for the proteins indicated. MDGA2 and SORT1 protein levels were quantified by densitometry, normalized to β-actin levels, and compared to respective controls (set to one arbitrary unit). n=3. Data represent mean ± SEM. *P* values were determined by 2-tailed unpaired Student's t-test. $*p < 0.05$, $**p < 0.01$, $***p < 0.001$.

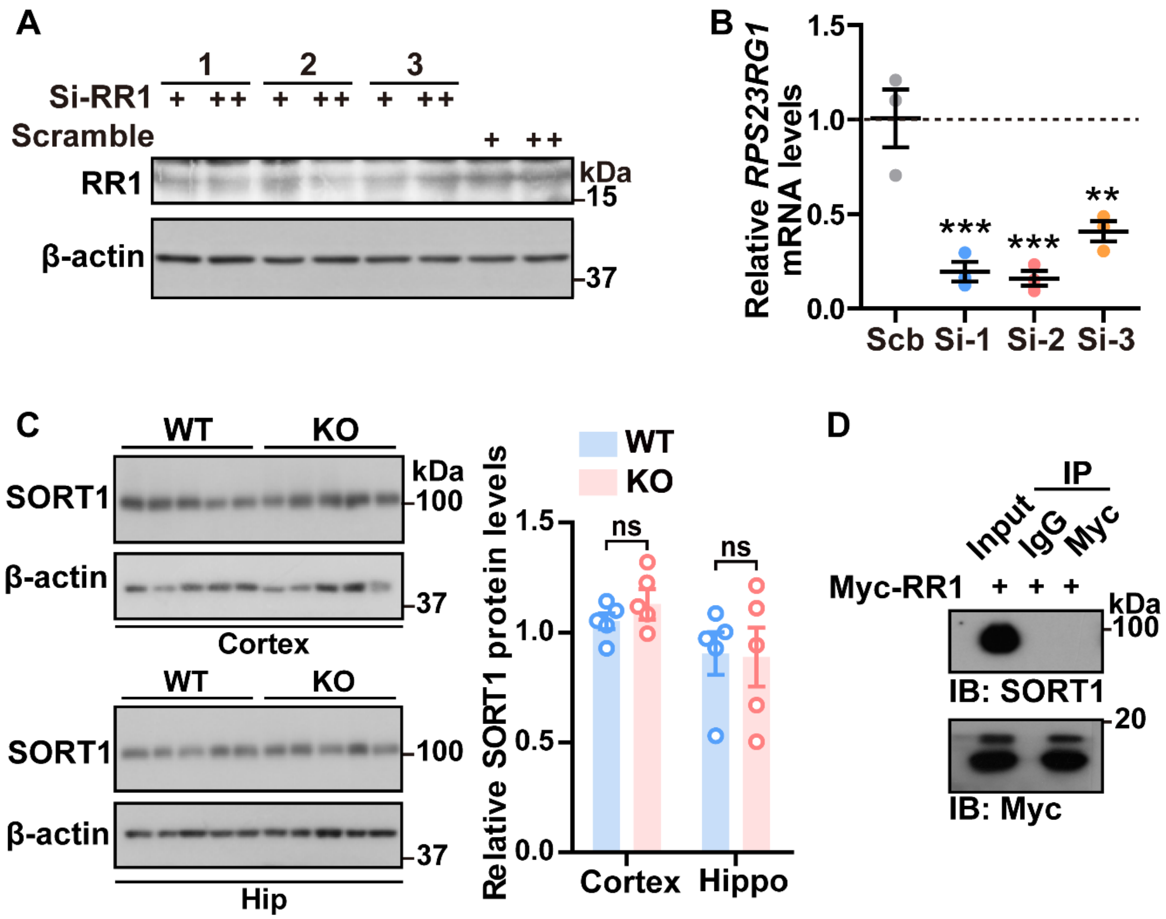

Figure S5. RPS23RG1 does not interact with and affect SORT1. (A) HEK293T cells were transfected with increased levels (+: 100 pmol; ++: 200 pmol) of Scramble (Scb) and RPS23RG1 (RR1) siRNAs (Si-1, Si-2, and Si-3) for 48 h. Equal protein amounts of cell lysates were immunoblotted for RR1 and β-actin. **(B)** HEK293T cells were transfected with Scb and various RPS23RG1 siRNAs for 48 h. *RPS23RG1* mRNA levels were determined by qRT-PCR, normalized to those of β-actin, and compared to those of controls (set to one arbitrary unit). n = 3. **(C)** Equal protein amounts of lysates from the cortex and hippocampus (Hip) of WT and *Rps23rg1* KO mice were immunoblotted for SORT1 and β-actin. SORT1 protein levels were quantified by densitometry, normalized to β-actin

levels, and compared to those of WT (set to one arbitrary unit). n=5. **(D)** HEK293T cells were transfected with Myc-RR1. Equal protein amounts of cell lysates were immunoprecipitated (IP) with IgG and an anti-Myc antibody and then immunoblotted (IB) for the proteins indicated. Data represent mean ± SEM. *P* values were determined by 2-tailed unpaired Student’s t-test. $***p < 0.001$, ns: not significant.

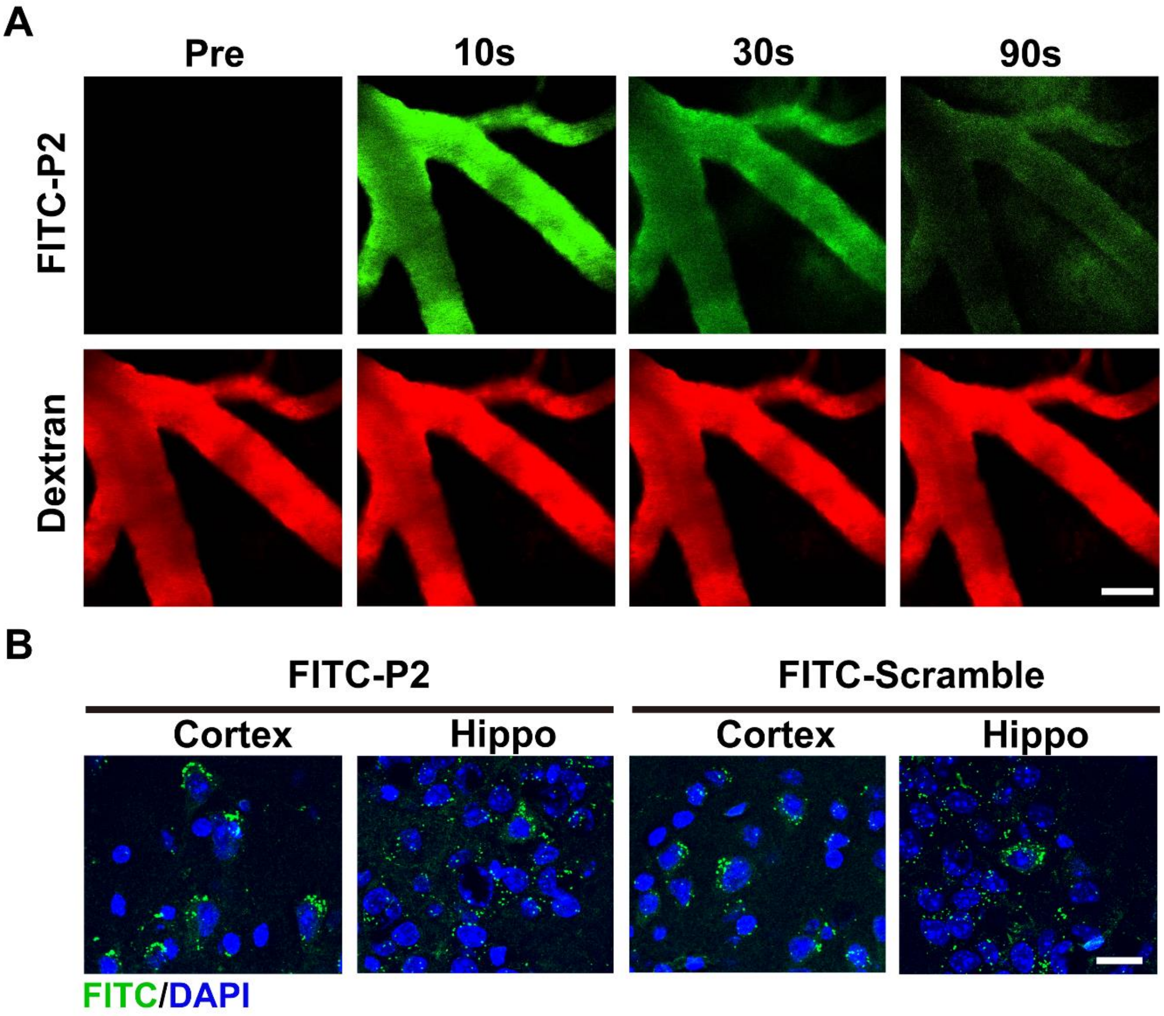

Figure S6. Assessment of the BBB permeability of peptides. (A) Four-month-old *Mdga2*$^{+/-}$ mice were first intravenously injected with Texas red-conjugated dextran (red) to visualize cortical vasculature, and then intravenously injected with FITC-labeled P2 peptide (green). Images were recorded for 90 s using two-photon imaging. Scale bar: 50 μm. **(B)** FITC-labeled P2 and scramble peptides (green) were intravenously injected into *Mdga2*$^{+/-}$ mice. Brain tissues were taken 12 h later to observe the distribution of the peptides in the hippocampus and cortex under a confocal microscope. The nuclei were stained by DAPI (blue). Scale bar: 20 μm.

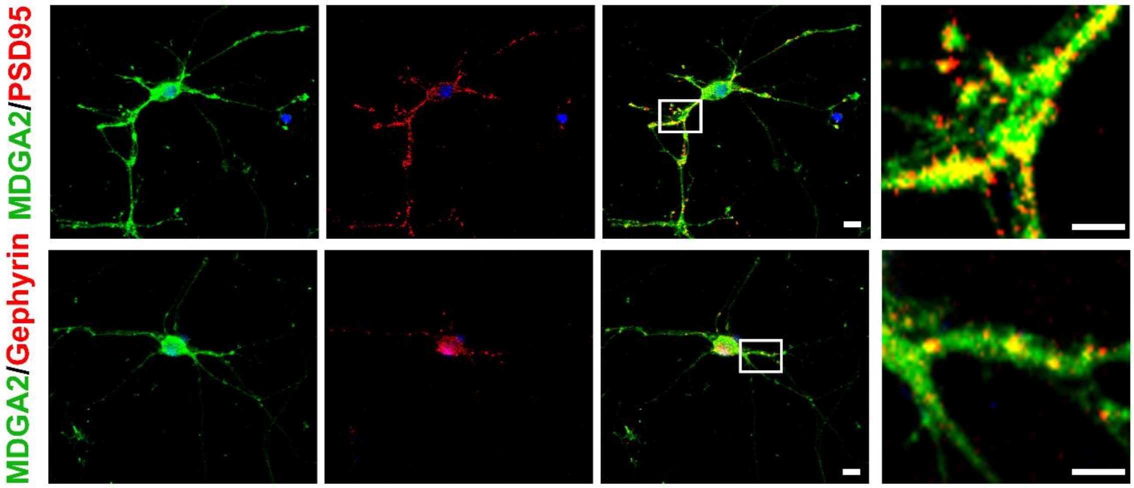

Figure S7. Distribution of MDGA2 in both excitatory and inhibitory synapses. Co-immunostaining of MDGA2 (green) with PSD-95 (red) or Gephyrin (red) in cultured mouse primary neurons at DIV14. Scale bars = 10 μm, and scale bars = 5 μm in enlarged images.
